# Pacemakers in Modern Cardiology and Their Transition From Traditional to Leadless Models

**DOI:** 10.7759/cureus.82182

**Published:** 2025-04-13

**Authors:** Yusuf Ali Serbetci, Shelly Jaryal, Ira Bhasin, Mohammad Elhassan, Amna Muslim, Parisa Aquilini, Likhitha Reddy A, David Tavera, Angela Pia Caicco, Manju Rai

**Affiliations:** 1 Internal Medicine, Lokman Hekim University, Ankara, TUR; 2 Medicine, Medical University of the Americas (MUA), Charlestown, KNA; 3 Internal Medicine, Kasturba Medical College, Manipal, IND; 4 Internal Medicine, Weill Cornell Medicine-Qatar, Doha, QAT; 5 Internal Medicine, Karachi Medical and Dental College, Karachi, PAK; 6 Internal Medicine, The Royal College of Surgeons of Ireland, Dublin, IRL; 7 Internal Medicine, Madras Medical College, Chennai, IND; 8 Surgery, Jose Felix Patino’s School of Medicine, Bogotá, COL; 9 Internal Medicine, Magna Grecia di Catanzaro, Calabria, ITA; 10 Biotechnology, Shri Venkateshwara University, Gajraula, IND

**Keywords:** cardiac rhythm management, cardiovascular devices, leadless pacemakers, minimally invasive technology, multicomponent pacing systems, traditional pacemakers, transvenous leads

## Abstract

Cardiac rhythm disorders are a major subset of cardiovascular diseases and continue to pose significant global health challenges. Pacemakers remain central to their management, with evolving technologies offering improved therapeutic outcomes. This narrative review traces the progression of pacemaker technology from traditional cardiac pacemakers (TCPs) to leadless cardiac pacemakers (LCPs), emphasizing key differentiators such as implantation techniques, complication profiles, and long-term clinical performance. While TCPs have proven effective, they are associated with issues like lead-related complications, infections, and mechanical failures. In contrast, LCPs, which are implanted via minimally invasive transcatheter approaches and lack transvenous leads, significantly reduce these risks, resulting in improved patient satisfaction and fewer hospitalizations. Although limited to single-chamber pacing and higher initial costs, LCPs may prove cost-effective over time due to lower complication-related expenses. This review synthesizes current evidence on safety, efficacy, and patient outcomes and explores recent advancements, including multicomponent systems, energy-harvesting mechanisms, and biocompatible materials. Many of these innovations are in preclinical or early clinical stages but hold promise for broader applicability and long-term sustainability. Despite challenges, the trajectory of pacemaker technology underscores its potential to revolutionize cardiac care, driven by increasing clinical adoption of leadless systems, evolving regulatory approvals, and a growing emphasis on equitable access and personalized management.

## Introduction and background

Cardiovascular diseases (CVDs) remain the leading cause of mortality worldwide. In 2021, CVDs accounted for approximately 20.5 million deaths globally, representing close to one-third of all deaths [1]. In the United States, heart disease continues to be the leading cause of death [2]. In 2022, 702,880 individuals succumbed to heart disease, equating to one in every five deaths [2-3]. These statistics underscore the critical importance of effective management strategies for cardiac conditions, including the utilization of pacemakers to address rhythm disorders.

Pacemakers have long been pivotal in managing cardiac rhythm disorders, transforming the treatment of conditions like bradyarrhythmias and heart block. These small, implantable devices have the remarkable ability to restore and maintain normal heart rhythms, significantly improving patients' quality of life and reducing mortality associated with arrhythmias [4]. Over the years, pacemakers have not only become integral to cardiology but have also exemplified the intersection of clinical need and technological advancement [5].

The evolution of cardiac pacing technology is a testament to innovation driven by the pursuit of better patient outcomes. Traditional cardiac pacemakers (TCPs), introduced in the mid-20^th^ century, relied on a pulse generator connected to the heart through transvenous leads [6]. While highly effective, these systems were not without limitations, including the risks of lead fracture, infection, and long-term durability concerns related to lead failure, battery depletion, and device-related complications [7]. Additionally, traditional models are often limited to single-chamber pacing in certain cases and may involve high costs associated with surgical implantation and maintenance [7]. In response to these challenges, significant strides have been made in device miniaturization, battery longevity, and the development of leadless cardiac pacemakers (LCPs). The advent of LCPs marks a paradigm shift, eliminating the need for transvenous leads and reducing complications associated with traditional designs [8]. These devices represent a new frontier in cardiac pacing, offering minimally invasive implantation and enhanced patient comfort.

This review aims to provide a comprehensive exploration of the advancements in pacemaker technology, focusing on the transition from traditional to leadless models. It delves into the historical development of pacemakers, examining the milestones that have shaped their current form and functionality. The scope of this review extends to a critical evaluation of the clinical efficacy, safety, and limitations of both traditional and leadless systems. By synthesizing current evidence, this review seeks to offer valuable insights into the practical and theoretical aspects of pacemaker technology, fostering a deeper understanding of its trajectory and future prospects.

## Review

Historical perspective

Like many technological advancements, the development of the pacemaker encountered numerous challenges that ultimately shaped the way cardiac arrhythmias are managed today. The journey toward cardiac pacing began in 1791 with Luigi Galvani’s groundbreaking experiments on frogs, which demonstrated the electrical nature of muscle contraction [9]. While Galvani’s discoveries did not directly lead to pacemaker technology, his work on bioelectricity laid the foundation for understanding how electrical impulses regulate cardiac function. This fundamental knowledge eventually contributed to the development of electrical stimulation for managing arrhythmias.

The concept of cardiac pacing materialized in the mid-20th century, driven by the pioneering efforts of Albert Hyman and Mark Lidwell [9]. They developed early cardiac pacing machines that, although rudimentary, demonstrated the potential of electrical stimulation in regulating heart rhythm. However, these early devices faced ethical and clinical skepticism, with concerns about their long-term effects, patient safety, and the notion of artificially altering heart function. Early pacemaker adoption was also hindered by regulatory uncertainty, as the medical community debated the risks and benefits of permanently implanting such devices.

A major breakthrough occurred in the 1950s with the invention of the transistor, enabling the creation of smaller and more efficient devices suitable for implantation. In 1952, Dr. Wilfred Bigelow and Dr. John Callaghan used cardiac pacing to prevent cardiac arrest during hypothermia-induced surgeries [10]. By 1958, the first successful pacemaker implantations for treating bradyarrhythmias were performed in Sweden [11]. In 1960, Dr. Wilson Greatbatch developed the first battery-powered, implantable pacemaker, marking a pivotal step in the evolution of this life-saving technology [12]. The initial battery lifespan of these early devices was approximately two years, necessitating frequent replacement surgeries [13].

The 1980s witnessed the introduction of implantable cardioverter-defibrillators (ICDs), which expanded the capabilities of pacemakers beyond treating bradyarrhythmias [12]. Unlike standard pacemakers, ICDs were designed not only to provide pacing but also to detect and terminate life-threatening tachyarrhythmias through electrical shocks. This advancement significantly improved arrhythmia management, particularly for patients at risk of sudden cardiac death. Additionally, the development of cardiac resynchronization therapy (CRT) broadened the role of pacemakers, offering biventricular pacing to improve outcomes in select patients with heart failure and ventricular dyssynchrony.

The early stages of pacemaker development were fraught with challenges. Initial devices were bulky and invasive, leading to complications such as bleeding, infections, and lead dislodgement during implantation [13]. The rigidity of early pacing wires further complicated procedures, increasing the risk of mechanical failure. Portability was another major limitation, as the first models were stationary and required external power sources, significantly restricting patient mobility and quality of life. By the 1960s, smaller, battery-powered pacemakers became available, with lifespans gradually extending to five to 10 years, reducing the frequency of replacement surgeries [14].

Additionally, early pacemakers lacked adaptability, offering only fixed-rate pacing without accommodating physiological variations, such as heart rate adjustments during sleep or physical activity. This limitation underscored the need for more advanced pacing algorithms, ultimately leading to the development of rate-responsive pacemakers capable of dynamically adjusting pacing based on the body’s demands [15].

Traditional cardiac pacemaker designs

**Figure 1:**
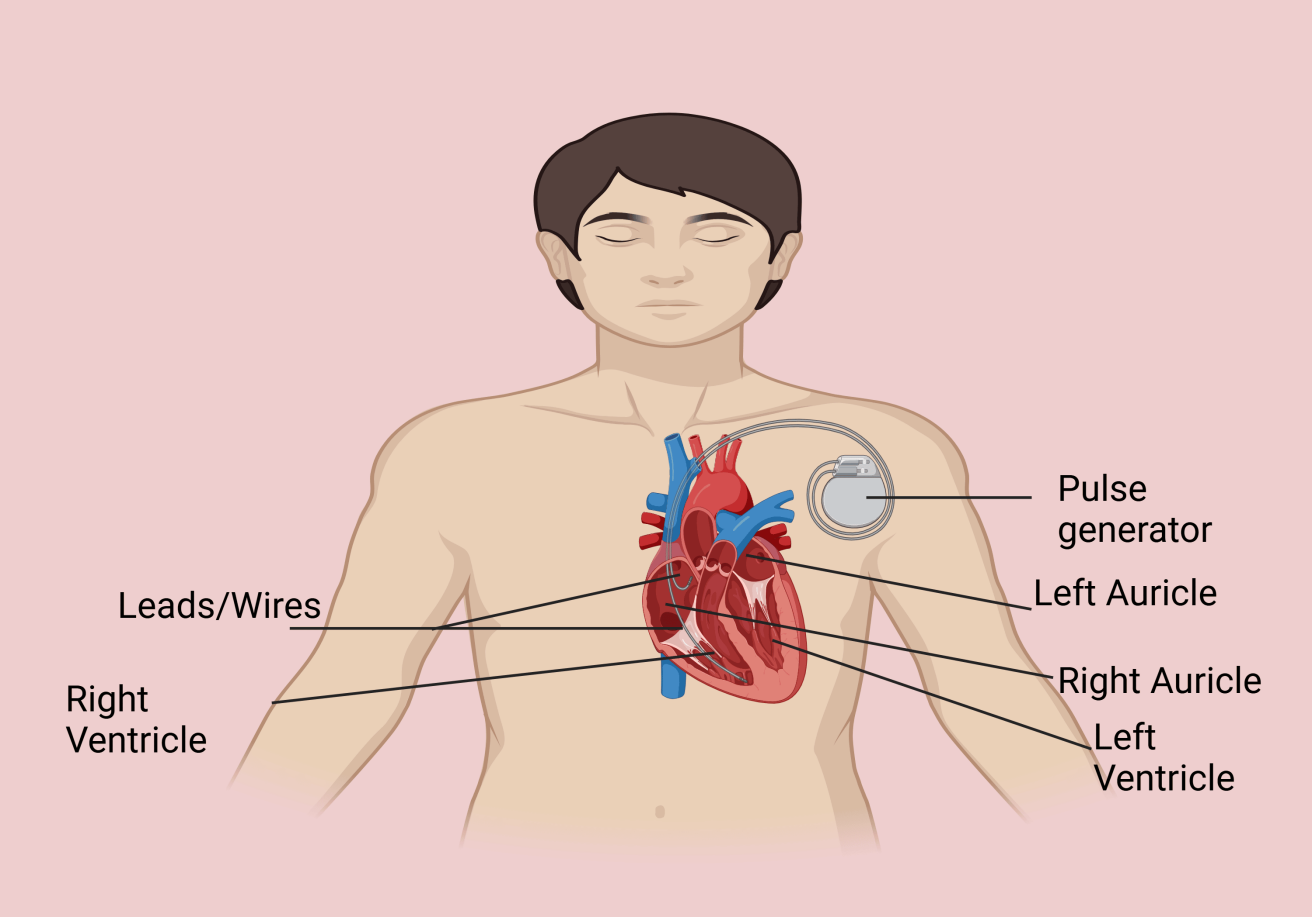
Traditional cardiac pacemakers This figure has been created by the author David Tavera, using BioRender.com (https://biorender.com).

Traditional cardiac pacemakers rely on well-established anatomical designs and working principles that have evolved over decades to address various cardiac conditions. A TCP consists of three key components: the pulse generator, leads, and electrodes. The pulse generator, typically implanted subcutaneously in the chest, houses a battery and circuitry to produce electrical impulses. Leads, which are thin, insulated wires, extend from the generator to the heart chambers. At their distal ends, electrodes are positioned on or within the myocardium to deliver the impulses directly to cardiac tissue (Figure 1).

Traditional cardiac pacemakers can be classified into three main types based on their lead placement and functionality (Table 1). Single-chamber pacemakers feature a single lead placed in either the right atrium or right ventricle and are primarily used for patients with atrial fibrillation and a slow ventricular response or those with sick sinus syndrome [16]. Dual-chamber pacemakers, on the other hand, utilize two leads, one in the right atrium and the other in the right ventricle, allowing them to mimic the heart’s natural atrioventricular synchronization. These devices are ideal for patients with atrioventricular block or sinus node dysfunction [17]. Biventricular pacemakers, also referred to as CRT devices, involve the placement of leads in the right atrium, right ventricle, and coronary sinus on the left ventricle’s surface. These are particularly effective for patients with heart failure and ventricular dyssynchrony, as they improve ventricular contraction efficiency and overall cardiac function [18].

**Table 1:** Comparison of single-chamber, dual-chamber, and biventricular pacemakers AV: atrioventricular; CRT: cardiac resynchronization therapy; LBBB: left bundle branch block; QRS complex represents the depolarization of the ventricles Source: [16-18]

Aspect	Single-chamber pacemaker	Dual-chamber pacemaker	Biventricular pacemaker
Pacing chambers	One chamber (either atrium or ventricle)	Two chambers (atrium and ventricle)	Three chambers (right atrium, right ventricle, left ventricle)
Indications	Bradycardia or atrial fibrillation with slow ventricular response	Sick sinus syndrome, AV block	Heart failure with dyssynchronous ventricular contraction (e.g., LBBB)
Mechanism	Stimulates either atrium or ventricle	Coordinates atrial and ventricular contraction	Resynchronizes the contraction of both ventricles
Benefits	Simple, cost-effective, fewer complications	Improves cardiac output by maintaining AV synchrony	Enhances ventricular efficiency and reduces heart failure symptoms
Limitations	Loss of AV synchrony if ventricle-paced	May not correct ventricular dyssynchrony	More complex implantation, higher cost
Programming	Simpler programming, fewer settings	More complex programming to optimize AV timing	Advanced programming for CRT
Battery life	Longest (eight to 15 years) due to simpler functionality	Moderate due to dual pacing	Shorter (five to 10 years) due to the increased activity of the three leads
Target patients	Patients needing minimal pacing	Patients needing synchronized atrial and ventricular pacing	Heart failure patients with dyssynchrony (e.g., LBBB or wide QRS complex)

Traditional cardiac pacemakers are primarily indicated for conditions like symptomatic bradycardia, atrioventricular blocks, and certain forms of heart failure. Additionally, newer indications have gained attention, such as pacing for neurocardiogenic syncope and hypertrophic cardiomyopathy, particularly in patients with left ventricular outflow tract obstruction who may benefit from strategic ventricular pacing [19].

Despite their benefits, traditional pacemakers are not without complications. Lead-related issues are among the most significant drawbacks, including lead dislodgement, fractures, venous obstruction, and infection [7]. Over time, fibrotic tissue formation around leads can also lead to pacing threshold increases, necessitating higher energy output and premature battery depletion. Additionally, lead extraction procedures can be complex and associated with significant risks [7]. To address these limitations, LCPs were developed. Leadless cardiac pacemakers are self-contained, battery-powered devices that are implanted directly into the heart via a transcatheter approach, eliminating the need for leads.

Advancements in pacemaker technology

Recent advancements in pacemaker technology have significantly enhanced device functionality, patient safety, and quality of life. One of the key areas of progress is miniaturization, which has enabled the development of LCPs and other compact devices [20, 21]. Traditional cardiac pacemakers required extensive surgical procedures for implantation due to their size and reliance on leads. However, miniaturized designs, such as LCPs, eliminate the need for leads and can be implanted directly into the heart via minimally invasive techniques [22, 23]. Devices like the Micra Transcatheter Pacing System (Medtronic, Minneapolis, MN) have demonstrated excellent safety profiles and comparable efficacy to conventional pacemakers while reducing risks associated with lead-related complications and infections [24, 25]. Additionally, advancements in battery technology have extended device longevity, with some modern LCPs offering battery life exceeding 15 years, minimizing the need for replacement surgeries [26].

Another critical innovation is the use of biocompatible materials, which enhance the safety and durability of pacemaker components. Modern pacemakers incorporate materials like titanium for casing, which is highly resistant to corrosion and well-tolerated by the body. Improvements in lead insulation materials, such as silicone and polyurethane, have reduced the likelihood of lead failure and prolonged device lifespan. Biocompatibility also plays a significant role in reducing the risk of device-related infections and inflammatory responses, which were common complications with earlier TCP designs [27]. Researchers are also exploring the potential of bioresorbable materials, which could allow for temporary pacing solutions (post cardiac surgery or transient heart block) without the need for device removal [28].

Wireless communication features represent another transformative advancement in pacemaker technology. Modern devices are now equipped with remote monitoring capabilities, allowing for real-time data transmission to healthcare providers. This innovation reduces the need for frequent in-person visits, enabling early detection of device malfunctions or changes in a patient’s condition. Wireless systems like the Medtronic CareLink Network provide continuous telemetry data, improving patient outcomes through proactive interventions [29]. Moreover, these systems enhance patient compliance and provide insights into pacing efficiency and battery status, streamlining clinical management [30]. However, remote monitoring also introduces challenges, such as data security concerns, reliance on external infrastructure, and the need for patient education on proper use. Ensuring secure data transmission and patient adherence remains a key focus in the widespread implementation of these technologies.

Ongoing research is also investigating energy-harvesting technologies to reduce reliance on battery replacements. Techniques such as piezoelectric energy harvesting (which converts mechanical cardiac motion into electrical energy) and kinetic energy harvesting have shown promise as potential self-sustaining power sources for pacemakers [28]. These approaches could eventually eliminate battery-related limitations, making pacemakers more sustainable and reducing the need for replacement surgeries.

Leadless pacemakers: a paradigm shift

**Figure 2:**
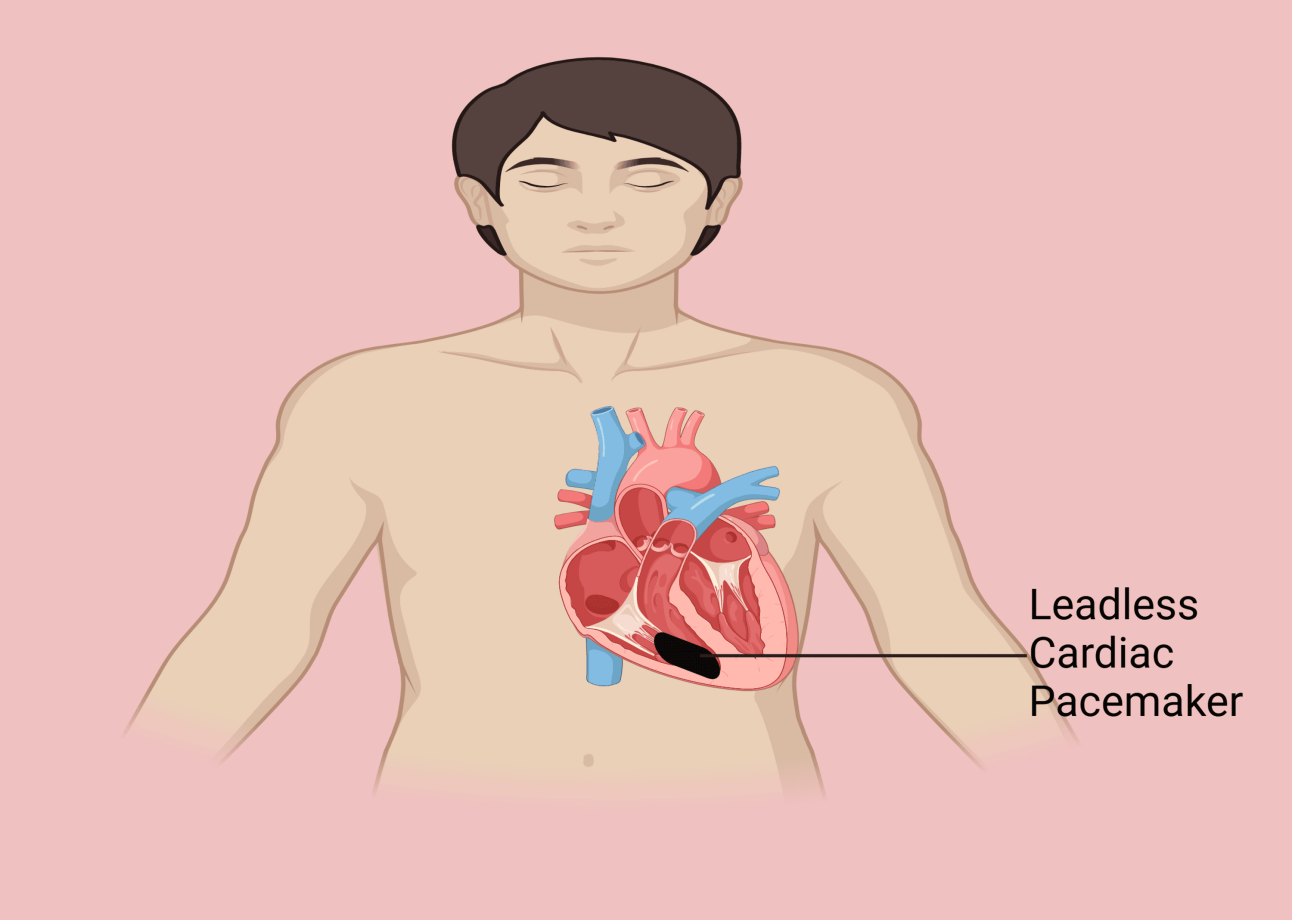
Leadless cardiac pacemakers This figure has been created by the author Ira Bhasin, using BioRender.com (https://biorender.com).

Leadless cardiac pacemakers embody a transformative innovation in modern cardiology, addressing several limitations associated with TCP systems. These devices integrate the pulse generator, battery, and electrodes into a single self-contained unit. Typically, they are small, capsule-like structures, measuring approximately 25-30 mm in length and weighing less than 2 grams (Figure 2) [31]. Leadless cardiac pacemakers are directly implanted into the right ventricular endocardium via a minimally invasive transcatheter approach, usually through the femoral vein [32].

Fixation mechanisms vary between manufacturers and are critical to device stability. Common strategies include elastic nitinol tines, as used in the Micra Transcatheter Pacing System, and active fixation via helix screws, such as those employed in the Aveir VR system (Abbott, Chicago, IL) [33]. These anchoring techniques securely attach the device to the myocardium, ensuring reliable contact for impulse transmission. Once deployed, electrical signals are delivered through electrode surfaces that lie in direct contact with the endocardium. Recent LCP models utilize advanced sensing and pacing algorithms to enhance responsiveness and adaptability to individual patient needs [34].

Traditional cardiac pacemakers require a subcutaneous pocket to house the pulse generator and transvenous leads for myocardial stimulation [35]. These systems, while effective, are associated with risks such as lead dislodgment, venous obstruction, and infections [21]. Leadless cardiac pacemakers, in contrast, eliminate these components, resulting in a smaller device and a simplified implantation process.

Functionally, current LCPs provide single-chamber ventricular pacing (VVIR mode), suitable for patients with sinus node dysfunction or atrioventricular block requiring minimal ventricular pacing [36]. While TCPs can offer dual-chamber or biventricular pacing, ongoing research and development aim to expand LCP capabilities. Devices such as the Micra AV (Medtronic), which incorporates an accelerometer to detect atrial contraction and allow atrioventricular-synchronous pacing, and Aveir DR (Abbott), designed as a modular dual-chamber system, are under active investigation and early clinical use [32].

The implantation technique for LCPs is less invasive, requiring venous access and fluoroscopic guidance for catheter-based delivery [36]. This contrasts with the surgical incisions and tunneling required for TCP systems. As a result, LCPs demonstrate reduced procedural complexity and shorter recovery times [37].

Leadless cardiac pacemakers offer numerous advantages over TCP systems, primarily by addressing complications associated with transvenous leads. Traditional leads are a common source of issues such as lead fracture, dislodgement, and venous thrombosis, all of which are entirely mitigated with leadless designs [21]. The minimally invasive transcatheter implantation approach further enhances patient outcomes by reducing surgical trauma, procedural time, and associated risks, particularly benefiting elderly or frail individuals. Additionally, LCPs significantly lower the risk of device-related infections by eliminating the need for subcutaneous pockets and transvenous leads, a notable advantage for patients with a history of sepsis or endocarditis [38]. Beyond medical benefits, LCPs also improve patient satisfaction by offering a device that is entirely internal, avoiding visible scars or the discomfort associated with a subcutaneous generator, making them especially appealing to younger patients or those concerned with aesthetics [39].

Clinical data support their efficacy and safety. The Micra Transcatheter Pacing System clinical trial reported a 96% implantation success rate, with a 48% reduction in major complications compared to traditional pacing systems [36]. Reported rates of device dislodgement are low, typically under 1.0%, while embolization events occur in <0.5% of cases [37].

Despite their numerous advantages, LCPs are not without limitations, which can impact their widespread adoption. One of the most significant challenges is their high upfront cost compared to traditional systems, making them less accessible, particularly in resource-constrained settings [40]. However, long-term cost-effectiveness may be favorable, as leadless systems are associated with fewer complications, lower infection-related hospitalization rates, and reduced re-intervention needs. These downstream savings are increasingly cited in support of broader LCP adoption [41].

Battery life, although improved in modern designs with lifespans of 10 to 12 years, remains a limiting factor, particularly since device retrieval and replacement pose challenges. Over time, endothelialization of the device surface, lack of standardized extraction tools, and the risk of device embolization or myocardial injury complicate retrieval attempts [22]. Consequently, many centers opt to implant a new device adjacent to the old one, a practice that may be suboptimal in younger patients with multiple device replacements anticipated over their lifetime. Another limitation is their restricted indication; LCPs are currently approved mainly for patients requiring single-chamber pacing, excluding those with advanced atrioventricular block or heart failure requiring cardiac resynchronization therapy [32]. Though dual-chamber and multi-site pacing options are being explored, these remain in development or early clinical phases. Furthermore, successful implantation of LCPs requires specialized training, fluoroscopy equipment, and institutional expertise, which may limit their availability in low-resource or rural centers [42].

Clinical evidence and outcomes

When evaluating safety, efficacy, and quality of life, the focus is on comparing leadless and transvenous pacemakers in terms of early or immediate complications, late complications, morbidity, and mortality. Among the LCPs discussed in reference studies, the Micra leadless pacemaker stands out, particularly when assessed under Coverage with Evidence Development (CED) criteria [43]. In this large-scale, real-world study, Micra recipients demonstrated a higher incidence of pericardial effusion and/or perforation (0.8%) compared to TCP recipients (0.4%) within the first 30 days. However, Micra patients experienced fewer overall device-related complications (2.7% vs. 4.0%) and a significantly lower need for system revisions at six months [43].

The safety and performance of Micra were further evaluated by Duray et al., who demonstrated a 48% reduction in major complications, leading to 47% fewer hospitalizations and 82% fewer system revisions in LCP recipients compared to Transcatheter Pacing System users over 12 months [37].

Another study evaluated the outcomes of Micra leadless pacemakers based on gender, considering comorbidities and complications [44]. Huang et al. reported that the outcomes of Micra leadless pacemaker implantation were similar across genders, demonstrating good procedural safety profiles and device functionality during long-term follow-up [44].

Overall, LCPs are considered safer than Transcatheter Pacing Systems in terms of pneumothorax, device dislodgement, reintervention rates, and overall complications. However, a meta-analysis by Shtembari et al. highlighted higher rates of pericardial effusion in the leadless pacemaker group as a notable exception [45]. Certain patient populations, such as those with chronic kidney disease (CKD), benefit uniquely from LCPs. Patients with CKD are at increased risk of device-related infections, vascular complications, and impaired wound healing, particularly when requiring hemodialysis access via central veins. Traditional transvenous leads can compromise vascular access essential for dialysis. Leadless cardiac pacemakers, being entirely intracardiac and inserted via femoral access, preserve central venous patency and reduce infection risk. In a retrospective study conducted by Panico et al., it was concluded that LCPs provide better survival and lower complication rates in patients undergoing hemodialysis compared to TCPs, making them the preferred option in this patient population [46].

Although the initial cost of some LCP models is higher, the reduced need for reinterventions and the lower risk of complications help offset their socioeconomic impact. A French longitudinal cohort study further supported the safety and efficacy of LCPs, concluding that they yield better outcomes in high-risk populations within one month of implantation [47].

Roberts et al. conducted a study to evaluate the acute performance of Micra pacemakers in real-world settings. They reported a high implantation success rate of 99.6% and a low complication rate of 1.51% within 30 days of implantation [48]. Another investigation focused on patients with CKD across various stages, assessing the operative and long-term safety and efficacy of LCP implantation in a real-world environment [49]. The findings indicated that LCPs demonstrated favorable safety and efficacy profiles in CKD patients, suggesting they should be considered the first choice for pacemaker implantation in this population [49, 50].

Boveda et al. examined two-year outcomes of LCPs versus TCPs in high-risk subgroups, based on the Micra Coverage with CED study. They found that patients with LCPs had lower reintervention rates and fewer complications compared to those with TCPs. High-risk subgroups included individuals with end-stage renal disease, CKD stages 4-5, malignancy, tricuspid valve disease (TVD), diabetes, or chronic obstructive pulmonary disease (COPD) diagnosed within 12 months prior to pacemaker implantation [51].

Key clinical trials have provided valuable insights into the comparison of leadless and transvenous pacemakers (Table 2). The Micra Transcatheter Pacing Study has been instrumental in understanding the safety and performance of Micra pacemakers. It concluded that LCPs are a safe alternative to transvenous systems, with most complications being minor and resolving without invasive intervention. The study’s safety endpoint was achieved within approximately two months, with no serious unanticipated adverse device events. Adverse events, primarily related to femoral access or transient dysrhythmias, were infrequent [52].

**Table 2:** List of some important studies reporting the key findings of leadless cardiac pacemakers.

Study	Key Findings
Duray et al. [37]	Patients with leadless pacemakers experienced 31% fewer chronic complications and a 38% lower reintervention rate compared to those with transvenous systems at 12 months follow-up.
Piccini et al. [43]	No significant differences in overall acute complication rates between the two systems. However, at 30 days, Leadless pacemaker recipients experienced a higher incidence of pericardial effusion and/or perforation. By six months, leadless pacemaker patients had lower overall complication rates compared to those with traditional pacemakers. Higher rates of pericardial effusion and/or perforation but lower rates of other device-related complications and the need for device revisions within six months.
Huang et al. [44]	The outcomes of Micra leadless pacemaker implantation were similar across genders, demonstrating good procedural safety profiles and device functionality during long-term follow-up.
Shtembari et al. [45]	Higher rates of pericardial effusion (2.65 times) are in notable exception, but generally much fewer complications (42% lower) in other parameters.
Panico et al. [46]	Leadless pacemakers provide better survival and lower complication rates in patients undergoing hemodialysis compared to traditional pacemakers which resulted in lead infections and central venous stenosis in such patients.
French Longitudinal [47]	Better outcomes in high-risk populations within one month of implantation (All-cause death of 8.3% as compared to 12.5% in conventional pacemakers).
Roberts et al. [48]	High implantation success rate of 99.6% and a low complication rate of 1.51% within 30 days of implantation.
Chronic Kidney Disease Study [49-50]	Leadless pacemakers demonstrated favorable safety and efficacy profiles in CKD patients, suggesting they should be considered the first choice for pacemaker implantation.
Boveda et al. [51]	Leadless cardiac pacemaker patients with malignancy, diabetes, tricuspid valve disease, and/or chronic obstructive pulmonary disease had lower rates of the combined outcome of device complications and select device-related reinterventions than TCP patients.
Micra Transcatheter Pacing Study [52]	Leadless pacemakers are a safe alternative to transvenous systems, with most complications being minor and resolving without invasive intervention.
Chami et al. [53]	Patients with leadless pacemakers experienced 31% fewer chronic complications and a 38% lower reintervention rate compared to those with transvenous systems at 24 months follow-up.

Piccini et al. conducted a contemporaneous analysis comparing outcomes between leadless and transvenous single-chamber ventricular pacemakers at 30 days and six months post implantation. The study observed no significant differences in overall acute complication rates between the two systems. However, at 30 days, LCP recipients experienced a higher incidence of pericardial effusion and/or perforation. By six months, LCP patients had lower overall complication rates compared to those with TCPs [43].

Duray et al. extended these findings in a 12-month study of the Micra Transcatheter Pacing System, demonstrating a 48% reduction in major complications for Micra patients compared to those with transvenous systems. This led to 47% fewer hospitalizations and 82% fewer system revisions in the LCP group [37].

Further insights into long-term outcomes were provided by a two-year follow-up study by Chami et al., which highlighted the efficacy and safety of the Micra leadless VVI pacemaker. Patients with LCPs experienced 31% fewer chronic complications and a 38% lower reintervention rate compared to those with transvenous systems. Adjusted all-cause mortality at two years was comparable between the two groups, underscoring the durability and reliability of LCPs [53].

Current challenges in leadless pacemakers

Several challenges have limited the widespread adoption and long-term use of LCPs. A primary drawback is their limitation to single-chamber pacing, significantly reducing their suitability for patients needing dual-chamber or multi-site pacing [54-55]. Although efforts are ongoing to develop multicomponent leadless pacing systems, these remain in experimental stages. Notably, the Avier™ dual-chamber leadless pacing system by Abbott is currently under clinical investigation, with the Avier DR i2i study (NCT05252702) evaluating its safety and efficacy in providing atrioventricular synchrony using two communicating leadless devices [56]. Similarly, Medtronic is developing a dual-chamber Micra system and investigating its feasibility in preclinical models. These innovations represent promising steps toward overcoming the current limitations of single-chamber leadless pacing. Synchronization between LCPs for dual-chamber pacing is another critical hurdle that must be addressed to broaden their applicability. Establishing reliable communication protocols between multiple LCPs implanted in different heart chambers is essential for achieving synchronized dual-chamber pacing [57]. Addressing technical challenges such as power consumption, signal interference, and maintaining stable connectivity within the dynamic cardiac environment is vital for realizing this advanced capability [58].

Battery longevity presents another significant constraint. Current LCPs offer a median projected battery life of five to 15 years, which is shorter than the ideal lifespan for implantable cardiac devices. This necessitates periodic replacements that carry inherent risks and complications [59]. Reducing current drain is crucial to support multi-chamber pacing systems with advanced control and communication features [60]. Additionally, the management of end-of-life LCPs remains undefined. Strategies such as placing a new device adjacent to the non-functional one or retrieving and replacing the old device have mixed feasibility and safety data [55,61]. The risks associated with multiple intracardiac devices, including cardiac perforation and device dislocation, remain a concern [62]. The absence of standardized protocols for managing non-functional LCPs underscores the urgent need for further research and guidelines to ensure patient safety and optimize outcomes.

Magnetic resonance imaging compatibility is another significant issue, as the integrated battery can interfere with diagnostic imaging, for instance, heating, device malfunction, and image artifacts, restricting its use in patients requiring frequent MRIs [11]. Some LCP models have conditional MRI compatibility. Furthermore, the significantly higher cost of LCPs compared to TCPs limits accessibility, particularly in resource-constrained settings. On average, LCPs are estimated to cost 1.5 to two times more than TCPs, with prices varying based on healthcare system reimbursement policies and regional procurement frameworks. In some healthcare systems, limited or conditional reimbursement for LCPs under CED programs further complicates widespread adoption. Despite these barriers, ongoing research aims to enhance the technology and reduce costs. Advances in battery technology and miniaturization techniques could result in more affordable LCPs. As adoption increases, economies of scale may further reduce production costs, improving accessibility for patients worldwide [63].

Procedural risks, including cardiac perforation, pericardial effusion, and vascular access complications, also pose challenges to their widespread use [62,64]. Additional issues such as retrieval difficulties, device dislocations, and the inability to integrate defibrillator systems limit their application in complex cardiac conditions [63]. Anatomical variations, such as dextrocardia and transposition of the great arteries, present further technical challenges in certain patients [65, 66]. These conditions alter the spatial orientation and position of cardiac structures, complicating catheter navigation, optimal positioning of the delivery sheath, and precise deployment of the leadless device. For example, in dextrocardia, the mirror-image anatomy can interfere with standard fluoroscopic guidance, while in transposition of the great arteries, abnormal alignment of the ventricles and outflow tracts can affect access and fixation. Moreover, as with any novel technology, a learning curve is associated with LCP implantation, which may initially result in higher complication rates compared to TCPs [67]. Studies suggest that proficiency in LCP implantation typically requires performing approximately 10 to 15 procedures, after which complication rates tend to decline significantly and outcomes become comparable to those of traditional systems. Early-phase data indicate a slightly elevated rate of cardiac perforation and vascular complications; however, these rates decrease as operators gain experience and adapt to the procedural nuances of leadless systems.

Future directions and innovations

Advancements in LCP technology continue to address current limitations, paving the way for transformative innovations. Key future directions include the development of emerging technologies, integration with other medical devices, and the incorporation of artificial intelligence (AI) for enhanced functionality.

Rechargeable batteries and energy harvesting systems represent promising solutions to extend the lifespan of LCPs. Rechargeable batteries powered via transcutaneous energy transfer (TET) could eliminate the need for periodic device replacements, reducing procedural risks and long-term costs. Preclinical studies have demonstrated the feasibility of TET, but technical challenges such as heat generation, low power transfer efficiency, and concerns regarding long-term biocompatibility must be overcome before clinical adoption [68].

Similarly, energy harvesting technologies utilizing kinetic, thermal, or electromagnetic energy generated by cardiac motion or surrounding tissues are under investigation. These remain mostly at the prototype or preclinical stage, as issues like low energy conversion efficiency, inconsistent power output, and device durability in physiological environments continue to pose hurdles [69].

The integration of LCPs with wearable monitors, implantable loop recorders, and defibrillators could revolutionize personalized patient care. Real-time communication between these devices may allow early detection of arrhythmias and device malfunctions. Although some companies, such as Medtronic and Abbott, are developing platforms for such integration, most efforts remain at the proof-of-concept or early clinical feasibility stage. Key barriers include secure data transmission, cross-platform compatibility, and regulatory approvals [70].

Artificial intelligence and remote monitoring systems also hold significant promise. Artificial intelligence algorithms could analyze LCP-generated data (e.g., ECG signals, heart rate variability, pacing trends) to predict arrhythmic events, optimize pacing thresholds, and identify patients at increased risk of complications. While AI has been applied in retrospective studies and small-scale trials, large-scale validation in the context of pacemaker optimization is still ongoing [71]. Remote monitoring platforms using AI are being tested for predictive alerts and real-time device oversight, and future iterations may support remote firmware updates for real-time performance enhancements [72].

Next-generation LCPs aim to address the limitations of single-chamber pacing. Multicomponent systems capable of dual-chamber or multi-site pacing are in development, such as Abbott’s Aveir™ DR system, currently under investigation in the Aveir DR i2i study (NCT05252702). These devices use inter-device wireless communication for synchronous pacing, aiming to replicate physiological cardiac rhythms. Major challenges include reliable synchronization, energy efficiency, and maintaining stable communication between components [73].

The high cost of LCPs remains a barrier to widespread adoption, particularly in low-resource healthcare settings. Several strategies are being explored to enhance affordability. These include pilot programs for subsidized LCPs, outcome-based reimbursement models, and tiered pricing based on regional income levels. Lessons may be drawn from cost-reduction strategies seen with generic drug-eluting stents and insulin pumps, such as local manufacturing, patent-sharing initiatives, and public-private partnerships. Ethical concerns regarding access to cutting-edge technology must be addressed through inclusive policies that promote affordability and equitable distribution, ultimately advancing global health equity.

## Conclusions

The shift from TCPs to LCPs represents a major advancement in cardiac rhythm management. Leadless cardiac pacemakers address critical limitations of TCPs by eliminating leads and subcutaneous pockets, thereby reducing infection rates, procedural complications, and hospitalizations. Real-world data have shown improved safety profiles and greater patient satisfaction. However, current limitations include high costs, restricted dual- or multi-chamber functionality, and challenges related to battery longevity and device retrieval. Multicomponent leadless systems, such as the Aveir™ DR, are undergoing early human trials, showing promise for broader physiological pacing but remaining years away from routine clinical use.

Future directions focus on overcoming these limitations through innovations in device miniaturization, energy harvesting, AI integration, and improved remote monitoring. Long-term safety, particularly regarding device retrieval and end-of-life management, requires standardized guidelines. To improve accessibility and reduce global disparities, strategies such as mass production, insurance coverage expansion, outcome-based pricing, and public-private partnerships should be explored. With continued innovation and thoughtful implementation, LCPs have the potential to transform the landscape of cardiac care and make advanced pacing technology more globally accessible.
